# Individual psychotherapy for cluster-C personality disorders: protocol of a pragmatic RCT comparing short-term psychodynamic supportive psychotherapy, affect phobia therapy and schema therapy (I-FORCE)

**DOI:** 10.1186/s13063-023-07136-z

**Published:** 2023-04-05

**Authors:** Martine Daniëls, Henricus L. Van, Birre van den Heuvel, Jack J. M. Dekker, Jaap Peen, Judith Bosmans, Arnoud Arntz, Marcus J. H. Huibers

**Affiliations:** 1 Arkin Mental Health Care, Amsterdam, The Netherlands; 2 Department of Clinical Psychology, VU University Amsterdam, Amsterdam, The Netherlands; 3 Department of Health Sciences, Faculty of Science, VU University Amsterdam, Amsterdam Public Health Research Institute, Amsterdam, The Netherlands; 4 Department of Clinical Psychology, University of Amsterdam, Amsterdam, The Netherlands; 5 Department of Clinical Psychology, Utrecht University, Utrecht, The Netherlands

**Keywords:** Personality disorders, Cluster-C, Individual psychotherapy, Effectiveness, Randomized clinical trial, Pragmatic trial, Affect phobia therapy, Schema therapy, Psychodynamic psychotherapy, Working mechanisms

## Abstract

**Background:**

Cluster-C personality disorders (PDs) are highly prevalent in clinical practice and are associated with unfavourable outcome and chronicity of all common mental health disorders (e.g. depression and anxiety disorders). Although several forms of individual psychotherapy are commonly offered in clinical practice for this population, evidence for differential effectiveness of different forms of psychotherapy is lacking. Also, very little is known about the underlying working mechanisms of these psychotherapies. Finding evidence on the differential (cost)-effectiveness for this group of patients and the working mechanisms of change is important to improve the quality of care for this vulnerable group of patients.

**Objective:**

In this study, we will compare the differential (cost)-effectiveness of three individual psychotherapies: short-term psychodynamic supportive psychotherapy (SPSP), affect phobia therapy (APT) and schema therapy (ST). Although these psychotherapies are commonly used in clinical practice, evidence for the Cluster-C PDs is limited. Additionally, we will investigate predictive factors, non-specific and therapy-specific mediators.

**Methods:**

This is a mono-centre randomized clinical trial with three parallel groups: (1) SPSP, (2) APT, (3) ST. Randomization on patient level will be pre-stratified according to type of PD. The total study population to be included consists of 264 patients with Cluster-C PDs or other specified PD with mainly Cluster-C traits, aged 18–65 years, seeking treatment at NPI, a Dutch mental health care institute specialized in PDs. SPSP, APT and ST (50 sessions per treatment) are offered twice a week in sessions of 50 min for the first 4 to 5 months. After that, session frequency decreases to once a week. All treatments have a maximum duration of 1 year. Change in the severity of the PD (ADP-IV) will be the primary outcome measure. Secondary outcome measures are personality functioning, psychiatric symptoms and quality of life. Several potential mediators, predictors and moderators of outcome are also assessed. The effectiveness study is complemented with a cost-effectiveness/utility study, using both clinical effects and quality-adjusted life-years, and primarily based on a societal approach. Assessments will take place at baseline, start of treatment and at 1, 3, 6, 9, 12, 18, 24 and 36 months.

**Discussion:**

This is the first study comparing psychodynamic treatment to schema therapy for Cluster-C PDs. The naturalistic design enhances the clinical validity of the outcome. A limitation is the lack of a control group for ethical reasons.

**Trial registration:**

NL72823.029.20 [Registry ID: CCMO]. Registered on 31 August 2020. First participant included on 23 October 2020.

## Administrative information

Note: the numbers in curly brackets in this protocol refer to SPIRIT checklist item numbers. The order of the items has been modified to group similar items (see http://www.equator-network.org/reporting-guidelines/spirit-2013-statement-defining-standard-protocol-items-for-clinical-trials/).

**Table:** 

Title {1}	Individual Psychotherapy for Cluster-C Personality Disorders: Protocol of a pragmatic RCT comparing Short-term Psychodynamic Supportive Psychotherapy, Affect Phobia Therapy and Schema Therapy (I-FORCE)
Trial registration {2a and 2b}.	NL72823.029.20 [Registry ID: CCMO]. Registered on 20-08-31 ICTRP: NL8545 [International Clinical Trials Platform]. Registered on 2020-04-20
Protocol version {3}	Version 3, January 2023
Funding {4}	This research is funded by Arkin Mental Health Care.
Author details {5a}	M. Daniëls, MSc: Arkin Mental Health Care, the Netherlands H.L. Van, PhD: Arkin Mental Health Care, the Netherlands B. van den Heuvel, MSc: Arkin Mental Health Care, the Netherlands Prof. J.J.M. Dekker, PhD: Department of Clinical Psychology, VU University of Amsterdam, the Netherlands J. Peen, PhD: Arkin Mental Health Care, the Netherlands Prof. J.E. Bosmans, PhD: Department of Health Sciences, Faculty of Science, Vrije Universiteit Amsterdam, Amsterdam Public Health research institute, The Netherlands Prof. A. Arntz, PhD: Department of Clinical Psychology, University of Amsterdam, the Netherlands Prof. M.J. H. Huibers, PhD: Utrecht University, Department of Clinical Psychology, and Arkin Mental Health Care, the Netherlands)
Name and contact information for the trial sponsor {5b}	H.L. Van, PhD: NPI, Arkin Mental Health Care, Domselaerstraat 128 1093 MB Amsterdam, the Netherlands, rien.van@arkin.nl
Role of sponsor {5c}	This funding source (Arkin Mental Health Care) had no role in the design of this study and will not have any role during its execution, analyses, interpretation of the data, or decision to submit results.

## Introduction

### Background and rationale {6a}

Personality disorders (PDs) are complex mental health disorders associated with low levels of quality of life, high health care costs, and poor prognosis. PDs are clustered in three clusters: A, B and C (DSM-V, [3]). Cluster-C PDs, including avoidant, dependent and obsessive-compulsive PDs, are among the most common mental disorders in the general population, with reported prevalence rates of 3–9% [64, 94]. See Table 1 for brief definition of the clusters. In clinical populations, this cluster of PDs is most prevalent [4] and is associated with high levels of burden, societal dysfunctioning, low quality of life [89] and chronicity of many other common mental health disorders [73, 80]. Research in the field of PD has, to date, mainly excluded the Cluster-C PDs and focused on borderline PDs. Also, Cluster-C PDs are generally not included in international guidelines for PDs [49]. This means that a large group of patients has been neglected, and knowledge on how to treat them is lacking.

**Table 1:** Brief definition of Cluster-A, B and C PDs

Cluster-A PDs (paranoid, schizoid and schizotypal PD) are considered to be the odd, eccentric disorders.	
Cluster-B PDs (borderline, narcissistic, histrionic, antisocial PD) are characterized by thoughts and emotions that are dramatic and overly emotional.	
Cluster-C PDs (avoidant, compulsive, dependent PD) are characterized by anxiety and fear (DSM-V, APA, 2013).	

Despite the scarcity of outcome studies, in routine clinical practice patients with (comorbid) Cluster-C PDs are frequently referred to mental health care and offered general treatments such as cognitive behavioural therapy or psychodynamic therapy. In order to obtain more evidence, this study compares the differential effectiveness of three individual psychotherapies for Cluster-C PDs: short-term psychodynamic supportive psychotherapy (SPSP), affect phobia therapy (APT) and schema therapy (ST).

#### Evidence from previous trials

To the best of our knowledge, only a few randomized clinical trials (RCTs) into individual treatment of Cluster-C PDs have been conducted [56]. In a large multicentre study (*N*=323), Bamelis et al. [11] compared the effectiveness of 50 sessions of schema therapy, open-ended clarification-oriented psychotherapy, and treatment-as-usual (TAU) in PD patients with mainly Cluster-C PD. They found ST to be superior to TAU and clarification-oriented psychotherapy on interview-based outcome.

The other four studies all consist of relatively small samples of 16 to 25 patients per treatment and considerable variations in doses and therapy forms. Emmelkamp et al. [33] found greater improvement in 20 sessions of cognitive psychotherapy in comparison with psychodynamic psychotherapy for avoidant PD (*N*=62). Svartberg et al. [93] found no difference between 40 sessions APT versus cognitive behavioural therapy for Cluster-C PDs (*N*=50). Hellerstein et al. [46] compared brief supportive psychotherapy with short-term dynamic psychotherapy (both 40 sessions) in 49 patients with mainly Cluster-C PDs, and found no significant differences between the therapies. Muran et al. [78] found significant differences only on some secondary outcomes that were not maintained at follow-up between 30 sessions psychodynamic therapy, brief relational therapy, and cognitive behavioural therapy for Cluster-C PDs (*N*=84). These few studies indicate that psychotherapies for Cluster-C are effective, with, except for the Bamelis et al. and Emmelkamp et al. studies, little differences between the various psychotherapy forms. However, the relatively small sample sizes of most studies limit the power to detect possible clinically relevant differences. No studies have been conducted comparing the effectiveness of SPSP, APT and ST for Cluster-C PD. Next, only the Bamelis study used an active control group, providing evidence for the unique therapeutic benefit of this therapy for Cluster-C PD. The other studies however did not use active control groups. This means the impact of the unique ingredients of these treatments for the Cluster-C PDs is still unclear.

### Explanation for the choice of comparators {6b}

#### Psychotherapies

In this study, we will compare schema therapy (ST), short-term psychodynamic psychotherapy (SPSP) and affect phobia therapy (APT). ST has gained interest worldwide in the treatment of PDs. Despite most research on ST for PDs has been focused on borderline PD, ST has also shown evidence in the treatment of Cluster-C PDs in clinical effectiveness, acceptability, and cost-effectiveness [9, 11]. Evidence for the effectiveness of ST has also been found for various anxiety and depression disorders [51, 72]. ST as delivered in our study is based on the Bamelis protocol [5, 6, 10].

A relatively wide range of group and individual psychodynamic therapies, long-term and short-term, have been evaluated mainly in mixed samples of personality pathology or borderline PD [37, 62]. In this study, we choose to study APT and SPSP. They are both time-limited psychodynamic therapies and include established interventions to address main problems related to Cluster-C pathology such as avoidance and dependence. In clinical practice APT is upcoming as a treatment for the Cluster-C PDs. It is developed by McCullough and has reformulated psychodynamic psychotherapy into a behavioural framework [53]. APT has been applied for treatment of various anxiety disorders, depression, and PDs. Empirical evidence of APT for (Cluster-C) PDs has been found in the earlier mentioned Svartberg study [93] and by Winston et al. [103].

SPSP is a psychodynamic psychotherapy that uses a supportive stance and techniques. It has been applied for depression with and without PD [60], focusing on depression in relation to inter- and intrapersonal patterns while taking into account etiologic longstanding personality vulnerabilities. SPSP has shown to be effective for depression in several RCTs [32]. In one of the first RCTs with SPSP [60], an effect on PD could be demonstrated independent from a concurrent improvement of depression. This is further corroborated in a current trial comparing SPSP to ST for patients with both depression and PDs [58]. The preliminary results indicate that these approaches could diminish both depression and PD pathology [59]. In our study, the SPSP protocol is slightly adjusted in particular to address personality pathology also in the absence of comorbid depression.

#### Process research

Understanding *why, how* and *for whom* a treatment works can offer insight how to ameliorate the treatment techniques and interventions to optimize its effectiveness [23]. This could be approached by studying predictors and therapy-specific and non-specific (common) working mechanisms. There are only a few studies on the potential predictors of differential outcome and the working mechanisms within Cluster-C PDs [1]. Lilliengren et al. [65] found that successful cases of psychotherapy for Cluster-C PDs were characterized by more engaged patients and unsuccessful cases by a more directive therapist stance. Some correlational process-outcome research has been done showing first support that change in some of the factors of the theoretical model of APT is related to outcome (for an overview see [53]). Analysis of the data of the Bamelis study suggests that two schema modes (i.e. the Healthy Adult and the Vulnerable Child) play a central role in the successive changes in the personality pathology [104]. The latter studies are based on differential theoretical backgrounds on psychotherapy for Cluster-C PDs. Process research based on a general Cluster-C theoretical model is lacking.

#### Potential mediators

In the research on mediators, general working mechanisms as well as specific working mechanisms are studied. In this study, specific working mechanisms for the three treatments SPSP, APT and ST are derived from the theoretical assumption about their effectiveness. In SPSP, the level of emotional and interpersonal insight is supposed to mediate outcome for personality factors. The assumption for APT is that restructuring of defence mechanisms (recognizing and learning to let go of dysfunctional defences), affect (ameliorating the experience and expression of adaptive emotions) and the self (ameliorating self-esteem) is mediating treatment outcome [96]. For ST, as mentioned before, Yakın et al. [104] have found that two schema modes, the Healthy Adult (integrated, wise and healthy functioning state) and the Vulnerable Child (state of anxiety, sadness, loneliness, or neglect/abuse), are central to the change process of personality pathology. Strengthening of the Healthy Adult mode and healing of the Vulnerable Child mode are hypothesized to reflect mechanism of change, not only in ST but also in other psychotherapies.

The influence of specific factors needs to be studied in the context of well-known common factors in psychotherapy. Numerous studies have demonstrated that the quality of the therapeutic alliance is a stable and relevant factor for change [38]. Zilcha-Mano [106] stresses the importance to differentiate between trait- and state-like components of alliance. The trait component refers to the patients’ general tendencies to form satisfying relationships. Clearly this also affects the relationship with the therapist. The state component describes the development of these tendencies or abilities of the patient through the interaction with the therapist. Thus the trait-like component could be considered as a general precondition for all therapeutic work. In contrast, the state-like component is assumed to be a potential discriminative curative factor, specifically related to either a therapy form or therapist. In addition, trait- and state-like alliance may play different roles in various therapy treatments. Comparing various psychotherapies allows us to disentangle potential differential effects of alliance in SPSP, APT and ST.

According to Kazdin [55], research on mechanisms of change needs to demonstrate a statistical association and a temporal relationship between treatment interventions, mediator and outcome, but also consistency and specificity of this mediator. Therefore, potential mediators and outcomes must be measured at multiple time-points during and after therapy, across the treatments [63]. These guidelines will be followed in the current study. Also, treatment interventions will be specified to explore the relationship between certain interventions, mediators and outcome.

#### Potential predictors

To gain more knowledge about what works for *whom*, potential predictors will be studied. We will investigate several general potential predictors (that may turn out to be moderators, i.e. differential predictors depending on treatment type): sociodemographic variables, subtype of PD, severity of psychiatric symptoms, severity of the PD. Next, after consultation of experts in the treatment of Cluster-C PDs, the following four specific potential predictors are defined.

- Childhood trauma: Childhood traumatic events are assumed at least partially to underlie the development of personality pathology, and adequately processing is helpful to reduce these problems [5, 6]. It has been identified as a negative predictor of treatment outcome for different psychiatric disorder [79],
- Level of autism traits: There is a high comorbidity and considerable overlap in symptoms between autism and PDs [69, 100]. Lugnegård et al. [70] found about half of the participants with an autism spectrum disorder (ASD) also met criteria for a PD, all belonging to Cluster-A or C. Effectiveness of psychotherapy for patients with PD and comorbid autism traits has not been studied, but Weston et al. [102] found lower effectiveness of treatment (cognitive behavioural therapy) of affective disorders within patients with ASD.
- Personality organization as defined by Kernberg [57]: The level of personality organization is defined by three domains: identity, primitive defence mechanisms, and reality testing. The severity of the personality pathology is ranging from neurotic personality organization to borderline personality organization to psychotic personality organization, the latter one being the most severe group of patients. Higher levels of personality organization are related to better treatment outcome [61]. Overall, in Cluster-C PDs, higher levels of personality organization were found than for Cluster-A or B PDs [29]. Until now, personality organization within Cluster-C patients and its impact on outcome has not been investigated.
- Vulnerable narcissism: Vulnerable narcissism is mainly manifested in the avoidant and obsessive-compulsive PD [76, 81] and is related to avoidant attachment and maladaptive, impulsive and avoidant ways of coping with stress [54]. No studies are available on the effectiveness of patients with Cluster-C PD and vulnerable narcissism, but vulnerable narcissism might be related to negative treatment outcome.

### Objectives {7}

The primary aim of this study is to compare the effectiveness and cost-effectiveness of the three individual treatments (SPSP, APT and ST) for Cluster-C PDs. The primary outcome of this superiority study is the severity of Cluster-C PD. We will compare the slopes over time of the three treatments from 0 to 12 months, with assessments of Cluster-C PD severity taking place at baseline, 1, 3, 6 and 9 months. Additionally, we will research differences between the outcomes of the three treatments at 18, 24 and 36 months follow-up. No hypothesis was formulated about the direction of a difference.

The second aim of this study is to explore working mechanisms by focussing on therapy-specific factors in the context of the contribution of common factors across psychotherapies. Selected therapy-specific factors are as follows: therapy-specific interventions; insight for SPSP; change in affect, defence style, perception of the self for APT; change in schema modes for ST. Working alliance is the non-specific factor being investigated.

The third aim is to identify general and specific predictors. Potential moderators will be studied exploratively. Firstly, we hypothesize that patients with relatively more childhood trauma will benefit more from ST than SPSP and APT, because of the use of imaginary rescripting, an evidence-based trauma processing technique [35, 83]. Secondly, we expect that patients with more severe personality pathology (e.g. borderline personality organization (Kernberg, [57]) will profit more from ST or SPSP than APT. This is because these therapies are developed and have proven their effectiveness for a broader range of PDs [60, 88], whereas APT is originally designed for the Cluster-C/neurotic population [25].

The relationship between specific treatment interventions, mediators and outcome will also be explored. Therapists will indicate per session which of the core interventions per treatment they have applied in their sessions. The hypothesis is that certain interventions will be stronger connected to mediators and outcome than others. For example, experiential techniques (imagery rescripting, multiple chair technique) for ST; adequate support for SPSP; facilitation of expression of emotions for APT.

With the results of this study, we aim to enlarge the evidence-based support for treatments of the Cluster-C population and contribute to the improvement of the quality of care for this vulnerable and scientifically relatively neglected group of patients [49].

### Trial design {8}

The I-FORCE study is a mono-centre RCT with three parallel treatments: (1) SPSP; (2) APT; (3) ST. The patient allocation ratio is 1:1:1. All treatments will provide 50 sessions, the first 32 sessions twice a week (4–5 months), the other 18 sessions weekly (4–5 months). I-FORCE is a pragmatic ecologically valid RCT, which means it will be conducted in the routine specialized clinical setting. Characteristics for a pragmatic trial are the use of minimal exclusion criteria and low barriers to patient participation. Waiving a comparison group with no treatment guarantees inclusion into an effective treatment and minimizes unwillingness to be randomized [35]. This means patients with a wide range of psychiatric comorbidity will be included in this study. Also, use of psychopharmacological medication is not a reason for exclusion.

## Methods: participants, interventions and outcomes

### Study setting {9}

Patients for I-FORCE are recruited from the referrals to the NPI, a Dutch mental health care centre specialized in treatment of PDs. Patients are preselected to the NPI by the central referral department of Arkin after screening for PD and previous treatment. The NPI is a specialist care provider for PD treatment and consists of three locations in Amsterdam (Centre-east, North and West) and one location in Amersfoort. The institute is specialized in both psychodynamic and ST and provides acknowledged clinical training programs for the registration of psychiatrists, clinical psychologists and psychotherapists in the Netherlands.

The treatments of the NPI are organized in three programs: a symptom and coping skills program, a focused personality change program (< 1 year treatment) and a structural personality change program (> 1 year treatment). Within these programs, various treatments are offered. Treatment indication is based on a process of shared decision making. All locations have a longstanding tradition in offering the study treatments SPSP, APT and ST.

### Eligibility criteria {10}^1^

#### Inclusion criteria

In order to be eligible to participate in this study, a patient must meet all of the following criteria:

- Primary diagnoses: DSM-V diagnosis Cluster-C PD or Otherwise specified PD with predominantly Cluster-C traits, operationalized as a minimum of 5 Cluster-C traits (see Table 2 for examples of Cluster-C traits)
- Age 18–65 years
- A written informed consent
- Dutch literacy
- The willingness and ability to participate in an individual treatment of 50 sessions in 1 year.

**Table 2:** Examples of Cluster-C traits

Avoidant PD:	
● Avoids occupational activities that involve significant interpersonal contact because of fears of criticism, disapproval or rejection.	
● Views self as socially inept, personally unappealing, or inferior to others.	
Dependent PD:	
● Needs others to assume responsibility for most major areas of his or her life.	
● Feels uncomfortable or helpless when alone because of exaggerated fears of being unable to care for himself or herself.	
Compulsive PD:	
● Shows perfectionism that interferes with task completion.	
● Is overconscientious, scrupulous, and inflexible about matters of morality, ethics or values (DSM-V, APA, 2013)	

#### Exclusion criteria

A potential subject who meets any of the following criteria will be excluded from participation in this study:

- (Subthreshold) Cluster-A or B PD
- Having received SPSP, APT or ST in the previous year
- Immediate intensive treatment or hospitalization is needed, e.g. acute suicidality
- Severe psychiatric disorder requiring priority in treatment (autism spectrum disorder, psychotic symptoms/disorder, bipolar disorder, severe substance use disorder)
- IQ <80. This will be assessed by the recruiting/intakers who carry out the eligibility assessments. When they suspect an IQ < 80 (mental retardation), standard procedure in the NPI is to conduct further diagnostic procedures on intelligence.

#### Therapists

All therapists in this trial are qualified psychologists or psychiatrists with additional training in SPSP, APT or ST for Cluster-C PDs. For ST therapists, a junior registration of the Dutch association of ST is requested (general course in ST and a minimum of 20 supervisions). For APT and SPSP therapists, a general course in APT or SPS is required, and in addition a minimum of 3 months supervision and approval of competence from the supervisor. Next, participating therapists will be trained in the specific Cluster-C protocols prior to the start of the trial. Biweekly peer supervision groups are organized to maintain therapy quality. We expect to include 20 therapists per treatment (SPSP, APT and ST). All therapists will receive a unique code.

## Interventions

### Intervention description {11a}

Patients are randomly assigned to SPSP, APT or ST. All treatments will consist of 50 sessions of 50 min and have a maximum duration of 1 year. The first 32 sessions will be offered twice a week, and after that, therapy will continue once a week (18 sessions).

ST is an integrative treatment approach combining cognitive behavioural, experiential, interpersonal and psychodynamic elements and techniques [105]. The therapy is based on the idea that dysfunctional schemas can develop when core fundamental needs in childhood were not met. It describes pathology in terms of modes; emotional states in which schemas are activated. The goal in ST is to develop a healthy adult mode by reducing dysfunctional coping modes, battling the punitive (self-criticizing) and demanding modes, teaching patients how to meet and be met in their emotional needs that were not met in childhood and processing of adverse childhood experiences, such as abuse and neglect [105]. In ST, use of experiential techniques has a central role in the therapy.

The ST-protocol for Cluster-C personality pathology [5, 6] will be used, except that the first 32 sessions are being offered in a twice instead of once a week frequency and the final sessions (bi)weekly instead of monthly. The protocol starts with a conceptualization phase of 6 sessions. Next, the main treatment phase focuses on experiential techniques (sessions 7 to 32). In the second phase, focus gradually shifts to behavioural change (sessions 33 to 47). The final three sessions are aimed at termination.

APT is based on the premise that internal conflicts about feelings—which is coined as affect phobia—underlie most psychologically based disorders. APT is a form of short-term dynamic psychotherapy (STDP), developed by McCullough and colleagues. APT integrates techniques from psychodynamic, cognitive behavioural and experiential therapies. Central in APT are the two triangles of Malan; the triangle of conflict and of person [74, 96]. The triangle of conflict represents the mechanics of phobic avoidance: defences and anxieties that modulate or block underlying adaptive feelings. The triangle of person represents the relationships where the pattern of conflict takes place. In the model, affects are viewed as primary motivators of behaviour and of change. The goal of the treatment is to gradually expose the client to adaptive feelings while preventing the defence and managing the anxiety until it decreases. For APT, some slight adjustments are made to equalize doses and intensity with the other two treatments in this study (Van Dam and Turksma, [111], internal publication).

SPSP is aimed to provide adequate support, fostering progression and countering regression by adequately gratifying unmet developmental needs in patients. Specific to SPSP is the distinction of eight levels of discourse (i.e. levels of insight attainable by the patient) that serve to structure and foster the therapeutic process. Therapy starts with levels 1 and 2, focusing on the patient’s physical and psychological symptoms and the influence of life circumstance. At the third and fourth level, the focus shifts to actual relational problems and patterns associated with the symptoms. In levels 5 to 8, if indicated and possible, the focus proceeds to the intrapsychic factors, for instance on how past relationships persist in the patient’s current life. At the lower levels, more supportive techniques are used, and at the higher levels, the therapist may use more interventions to facilitate insight, such as confrontation or clarification. The aim is to address the level that is necessary for the patient. The SPSP protocol is slightly adjusted in particular to address the Cluster-C pathology in the absence of comorbid depression (Van et al., [110], internal publication).

At our clinical site (NPI), SPSP, APT and ST are regularly delivered treatment options and supervisors and experienced clinicians are available. Treatment indication is normally performed by the procedure of shared decision making, in which the most suitable treatment (according to the diagnostic team) is presented to the patient, who makes the final choice. New therapists will be trained according to the registration demands of these therapies. In line with recent research showing higher frequency of therapy sessions to be associated with better outcome [18, 22], psychotherapy will be offered twice a week in the first 4-5 months and once a week the second part.

### Criteria for discontinuing or modifying allocated interventions {11b}

Patients can leave the study at any moment for any reason if they wish to do so, with the possibility of finishing the treatment (research dropout). Also, the therapist or the investigator can decide to withdraw a subject from the study for urgent (medical) reasons. Discontinuing treatment is possible in case of a severe psychiatric disorder requiring priority in treatment, deterioration/worsening of the disorder and practical reasons of insufficient commitment/motivation (treatment dropout). It is not possible to switch between different treatments (SPSP, APT and ST) in the trial. If there is a need for further treatment, the patient is offered a regular treatment at the NPI or will be referred.

### Strategies to improve adherence to interventions {11c}

All therapists are attending peer supervision groups at a minimum frequency once every 2 weeks. In every peer supervision group, an adherence attendant is installed to improve adherence to intervention protocols. Regular meetings between the adherence attendants and the researchers take place. In addition, all deviations from the protocol have to be announced and, if necessary, will be discussed in the research committee. The research committee consists of the coordinating researcher, a clinical expert from every treatment (SPSP, APT and ST), a psychiatrist/clinical researcher and a research assistant. They are not among the authors of this protocol. The research committee meets biweekly. Finally, all sessions will be audiotaped and adherence and competence checks will be performed on a selection of the tapes (a random sample of 20% of the treatment sessions).

### Relevant concomitant care permitted or prohibited during the trial {11d}

Care as usual will be delivered considering the prescription of medication. Use and change of medication will be monitored and analysed as a running covariate. Extra health care costs are incorporated in the cost-effectiveness analysis. In view of the long duration of the interventions and the pragmatic design of the trial, some psychotherapeutic co-interventions are allowed with a maximum of 10 sessions and after approval by the research committee. These are as follows: Eye movement desensitization and reprocessing, in case of comorbid post-traumatic stress disorder, booster sessions of motivational interviewing to prevent relapse in substance abuse and family or couples therapy in case of severe relational problems. All these co-interventions are monitored and sensitivity analyses will be done with these variables as covariates.

### Provisions for post-trial care {30}

Patients who drop out from treatments will be referred for regular clinical care. During the follow-up period, no specific interventions are offered. Patients are referred to their general practitioner if clinically necessary. Patients can apply for additional treatment 6 months after treatment termination (18 months after start treatment) with a referral from their general practitioner. In case of additional treatment, patients will not return to the same therapist.

## Outcomes {12}^2^

### Assessment and outcome measures

After the informed consent has been signed, baseline assessment is being conducted. Assessments include computer-based questionnaires and semi-structured interviews. All assessments will be administered in Dutch. To ensure quality of data collection of the semi-structured interviews, all assessors will be trained and supervised. Apart from the outcome measures described below, the demographics and other predictors are being assessed at the baseline measurement. An overview of the instruments at each assessment point is provided in Table 3.

**Table 3:**
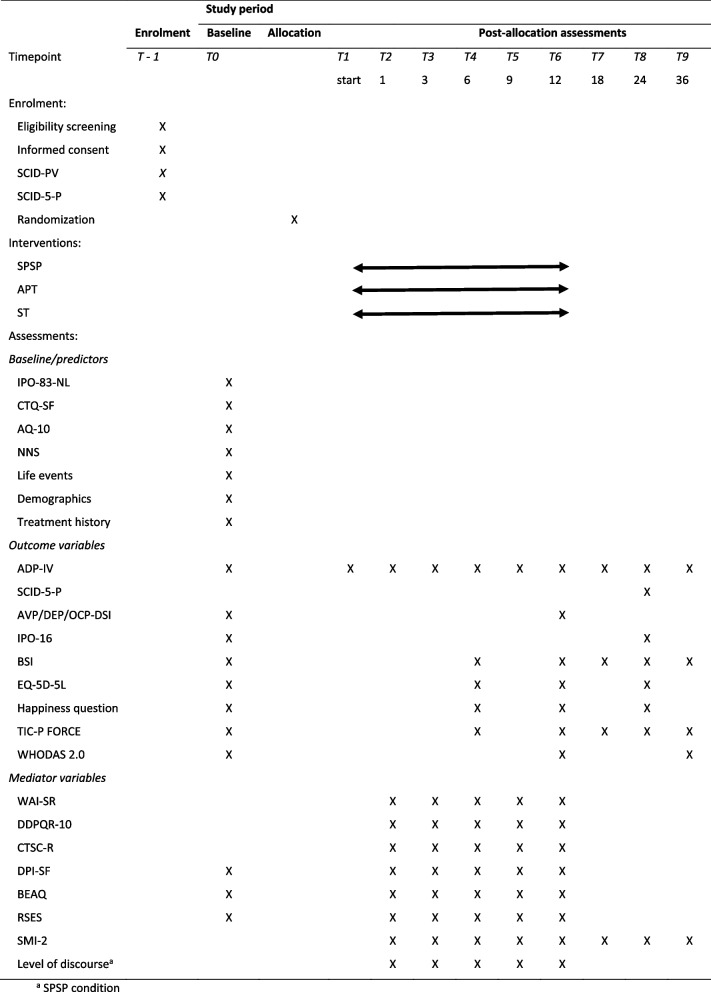
Overview of study design, all instruments and their time of assessment

#### Primary outcome measure

- Assessment of DSM-IV Personality Disorders questionnaire (ADP-IV, [87]). The primary outcome is the severity of Cluster-C personality pathology, measured by the ADP-IV. The ADP-IV is a self-report questionnaire, assessing DSM-IV PD criteria. Patients indicate on a 7-point Likert scale to what degree PD criteria hold for them, ranging from 1 (‘not at all’) to 7 (‘completely’), and whether they experience distress from it (on a range from 1—not at all to 3—definitely). Item construction of the ADP-IV allows for both dimensional and categorical diagnostic evaluation [87]. Adequate internal consistency, validity and reliability were shown consistently in previous studies [30, 85].

### Secondary outcomes

#### Personality functioning

- The Structural Clinical Interview for DSM-5 Personality disorders (SCID-5-P, [7]). This Dutch version of the SCID-5-P is used for diagnosing PDs at assessment and at 1-year follow-up. The SCID-5-P replaced the SCID-II. The exact reliability and validity of this version of the SCID-5-P is still unknown. Previous research, however, has shown adequate to good interrater reliability and test-retest interrater reliability of the original SCID-II, the Dutch version and translations of the SCID-5-P in other languages [42, 66, 71, 90]. Assessment using the SCID-5-P will be guided by items previously affirmed by the patient on SCID-5-PV (Structured Clinical Interview for DSM-5 Personality Questionnaire), a self-report questionnaire screening for PDs that will be completed at intake. Items not affirmed on the SCID-5-PV will be assumed to be true negatives; however, if a clinician has reason to believe these are false negatives, such items will be assessed. This method is in accordance with instructions for using the SCID-5-P and enables the assessment of PD symptoms to be based upon self-report combined with a structured clinical interview.
- Avoidant Personality Disorder Severity Index (AVPDSI) Dependent Personality Disorder Severity Index (DEPDSI), Compulsive Personality Disorder Severity Index (OCPDSI). These semi-structured interviews are developed to assess the frequency and severity of manifestations of the DSM-5 criteria of avoidant, dependent and compulsive PDs. For patients with a main diagnosis otherwise specified PD with predominantly Cluster-C traits, a personalized selection of the Cluster-C traits derived from the AVPDSI, DEPDSI and OCPDSI is made. These interviews have excellent interrater agreement (ICC>.90) and internal consistency (Cronbach *a*>.90) [44]. The interviews are modelled after the Borderline Personality Disorder Severity Index (BPDSI), measuring the severity of BPDs. The BPDSI is a reliable and valid instrument, suitable for use as an outcome measure [8, 43]. Total scores of the AVPDSI, DEPDSI and OCPDSI consist of a total sum of the average symptom scores per subsection of the interview, an average burden score and an average impact score. The scores on the instruments are converted into one severity score by standardizing the raw scores (see Groot et al. [44] for a description of the calculations per interview). To ensure quality of data collection of the AVPDSI, DEPDSI and OCPDSI, assessors will be trained, and all measurements will be audiotaped.
- Inventory of Personality Organisation Short Form (IPO-16-NL, [14]). The IPO-16-NL is the Dutch short version of the IPO-83 [21]. Norm scores of the German version are available and psychometric evaluation has shown good internal consistency, reliability, validity and confirmed a one factor structure of general personality dysfunction [107, 108]. The total score on the 16 items represents a dimensional measure of global severity of personality pathology according to Kernberg’s object-relationship framework.

#### Psychiatric symptoms

- Brief Symptom Inventory (BSI, [26]). The BSI is a 53-item self-report instrument that will be used to measure general psychological distress. The answers are scored on a 5-point Likert scale. It is derived from the SCL-90-R and has demonstrated it to be an acceptable short alternative of its longer version [24].

#### Quality of life, happiness and psychosocial functioning

- Quality of life (EQ-5D-5L, [48]). Quality of life is measured using the EQ-5D-5L. This self-report questionnaire assesses general quality of life using five domains: mobility, self-care, usual activities, pain/discomfort and anxiety/depression. Each dimension has 5 response levels: no/slight/moderate/severe/extreme problems. The Dutch norm scores will be used for calculating the mean EQ-5D utility values [99]. Psychometric study of the EQ-5D-5L has shown this version to be a valid and reliable extension of the three-level system [48, 52].
- Happiness Question [98]. The Happiness Question is added as a single question on general happiness in the months prior to the assessment and is scored on a 7-point Likert scale. This scale consists of the following verbal descriptions of different states of happiness: (1) completely unhappy, (2) very unhappy, (3) fairly unhappy, (4) neither happy nor unhappy, (5) fairly happy, (6) very happy, (7) completely happy. Dutch norms are available. For a single happiness item, high test-retest reliability (*r* = .86) and good concurrent, convergent, and divergent validity have been reported. The Happiness Question [28] has excellent sensitivity to change for patients with borderline PD who were treated with group ST.
- World Health Organization Disability Assessment Schedule (WHODAS 2.0, [95]). Psychosocial Functioning and Participation is administered with the WHODAS 2.0, a general measure of functioning and disability in major life domains, including understanding and communication, getting around, self-care, getting along with others, life activities and participation in society.

#### Costs

- Treatment Inventory Cost in Psychiatric Patients (TiC-P, [97]). Societal costs are assessed using a specifically adapted version of the TiC-P. The TiC-P FORCE is a 14-item self-report questionnaire to assess health care costs (part I) and costs resulting from productivity losses (part II) associated with psychiatric disorders. In part I, the number of contacts with different health care providers over the last 6 months is assessed. Part II consists of items regarding absenteeism from paid and unpaid work and presenteeism (i.e. reduced productivity while at work) in the last 6 months.

#### Potential mediators

Potential mediators being measured are divided in potentially general working mechanisms and therapy-specific working mechanisms. For the general or common working mechanisms, the following instruments are selected:

- Working Alliance inventory-short revised (WAI-SR). The WAI-SR is a 12-item self-report measure of the therapeutic alliance that assesses three key aspects of the therapeutic alliance: (a) agreement on the tasks of therapy, (b) agreement on the goals of therapy and (c) development of an affective bond. The WAI-SR demonstrated good psychometric properties [77].
- Difficult doctor-patient relationship questionnaire (DDPRQ-10, [45]). The DDPRQ-10 is a 10-item self-report questionnaire for therapists measuring the therapeutic relationship by investigating the extent to which patients are experienced as frustrating or difficult in the therapeutic relationship by their doctor or therapist. Five items are about the therapist’s subjective experience (e.g. “Do you find yourself secretly hoping that this patient does not return?”). Four items are quasi-objective questions about the patient’s behaviour (e.g. “How time consuming is care for this patient?”). One item combines elements of the patient’s behaviour and the therapist’s response (“To what extent are you frustrated by this patient’s vague complaints?”). The items are scored on a 6-point Likert scale. A high score reflects a high level of therapist frustration. In the study of Spinhoven et al. [91], the internal consistency of the DDPRQ-10 was .79.

In addition, instruments measuring potential working mechanisms specific to SPSP, APT and ST therapy are administrated in all three treatments.

For ST, the Schema Mode Inventory 2 (SMI-2, [12]) was selected to measure the schema modes:

- The SMI-2 is a modified version of the SMI-1 self-report questionnaire [68]. It consists of 143 items on 18 schema modes that are scored on a 6-point Likert scale. It measures the extent to which dysfunctional as well as functional schema modes are present. Its subscales have satisfactory to high internal consistency (Cronbach’s *α* ranges from .79 to .96), and it is considered to be a useful instrument for assessing modes [67]. Newly formulated modes proved to be appropriate for histrionic, avoidant and dependent PD. In line with Yakın et al. [104], the Vulnerable Child and Healthy Adult mode will be analysed. The Avoidant Protector and Impulsive Child will be analysed exploratory. At baseline, the complete SMI-2 will be administered. A shortened version with the modes relevant for Cluster-C PDs is used for the repeated measurements.

For SPSP, the level of emotional insight and level of discourse are chosen and operationalized in:

- Client Task-Specific Change Measure-R (CTSC-R, Watson et al: Client task-specific change measure–revised, unpublished)*.* This is a 16-item client self-report on a 7-point Likert-type scale, designed to measure the extent to which clients are able to identify changes, or newly acquired insight associated with particular sessions. A total score on the scale provides an index of client change following the session. Total scores of five or higher are indicative of moderate to high amounts of self-perceived change. The instrument is validated by Watson et al. [101] and showed good psychometric qualities with high internal consistency and item total correlations. Factor analysis showed the instrument comprises two factors, one dominant factor conceptualized as behaviour change and a second minor factor conceptualized as awareness and understanding.
- Level of discourse. The dominant level of discourse will be reported by the therapist at 1, 3, 6 and 9 months and at end of treatment, ranging from level 1 focusing on physical and psychological symptoms to level 8 focusing on the manifestation of the problems in the patient-therapist relation. This therapist-report scale is developed by Kool et al. [58].

For APT, restructuring of defence mechanisms, affect and self are chosen and operationalized in:

- Developmental Profile Inventory-Short Form (DPI-SF, [82]).The DPI is developed to assess psychodynamic personality functioning. It consists of nine subscales of developmental levels of psychodynamic functioning covering three domains: Self, Interpersonal Functioning and Defence/coping style. In this study, the DPI-SF consisting of the problem solving behaviour is being used to measure adaptive and non-adaptive defence styles. Internal consistencies of subsequent subscales were fair to good, ranging .71 to .91 in healthy controls and .67 to .88 in the patient sample. Mean corrected item total correlations were good, ranging .30 to .50. Test-retest reliability was good to excellent, with median intraclass correlation coefficient levels of .86 in healthy controls and .81 in the patient sample. The DPI also discriminated between patients and healthy controls in a meaningful way. Correlational analysis supported the distinction in a primitive and neurotic maladaptive cluster, and healthy adaptive cluster.
- Brief Experiental Avoidance Questionnaire (BEAQ). The BEAQ is a 15-item self-report measure. It is the shortened version of the 62-item Multidimensional Experiential Avoidance Questionnaire (MEAQ, [41]), measuring experiential avoidance. Items are scored on a 6-point Likert scale. A high score reflects a high level of experiential avoidance. Gámez and his colleagues have developed the short version BEAQ, selecting the 15 best performing items of the MEAQ [40]. Initial validation of the BEAQ has demonstrated good psychometric qualities. The psychometric qualities of the Dutch BEAQ have recently been studied by Slagter F, Topper M, Kamphuis JH, Nugter A: Measuring experiential avoidance: psychometric properties of the Dutch multidimensional experiential avoidance questionnaire, in preparation.
- Rosenberg self-esteem Scale (RSES). The RSES will be used to assess self-esteem. It is a widely used 10-item Likert scale self-esteem measure. Items are answered on a 4-point scale—from strongly agree to strongly disagree—measuring positive and negative feelings towards the self [84]. The Dutch version of the RSES is found to be a one-dimensional scale with high internal consistency and congruent validity and a Cronbach’s alpha of .89 [39].

For all three treatments, a self-report treatment integrity checklist is developed:

- I-FORCE Treatment Intervention List (I-FORCE-TIL)*.* After each therapy session, therapists will indicate which interventions they used in that session on a 30-item intervention list. This list has been developed by the authors of this study (Daniëls and Van den Heuvel, [109], internal publication), indicating the core interventions per treatment (SPSP, APT and ST) on a dichotomous scale (yes/no). Aim of this assessment is to register the applied treatment procedure. Reliability and validity checks will be performed on the I-FORCE-TIL.

#### Potential predictors and moderators

The selection of potential predictors or moderators to be examined was determined in consultation with experts:

- Inventory of Personality Organization (IPO-83-NL, [50]). The IPO is a self-report instrument consisting of 83 items on a 5-point Likert scale, based on Kernberg’s structural model of personality organization [21]. The Dutch version of the IPO has three main scales (Identity Diffusion, Primitive Defence and Reality Testing) and two supplementary scales (Aggression and Moral Values). The IPO-83-NL has good reliability and validity and appears to be a useful instrument to measure general personality pathology [15].
- Childhood Trauma Questionnaire-Short Form (CTQ-SF, [17]). This self-report measurement assesses childhood trauma. The short form was developed from the original 70-item version [16] and consists of 28 items measuring physical, sexual and emotional abuse and physical and emotional neglect. Its reliability and criterion-related validity have been established [17]. A recent study in the Netherlands confirmed its five-factor model [92].
- Nederlandse Narcisme Schaal (NNS, [34]). The NNS measures three dimensions of narcissism: overt (‘centrifugal’) narcissism and covert (‘centripetal’) narcissism and isolation. The questionnaire consists of 35 items with a 7-point Likert scale. Covert narcissism is hypothesized to be present in some patients with a Cluster-C PD and could possibly influence the outcome. The construction of this subscale is based on the Dutch translation of the hypersensitive narcissism scale [47] and consists of 11 items, with good reliability (Cronbach’s alpha .82). Dutch norms are available, although further research is necessary.
- Autism Spectrum Quotient short form (AQ-10). The AQ-10 Adult is derived from the original 50-item AQ [2], by a selection of the 10 items with the best discriminant validity. The questionnaire consists of 10 statements with for every statement four response options: strongly agree, slightly agree, slightly disagree, strongly disagree. At a cut-point of 6, sensitivity was .88, specificity was .91 and positive predictive value was .85.

#### Therapist adherence and competence

To assess treatment integrity and therapist competence, all individual treatment sessions will be audio recorded. A random sample of 20% of the treatment session recordings will be rated by independent trained judges blind for treatment, using structured measures during as well as post-hoc. For SPSP, adherence will be rated by the SPSP Adherence Scale that was developed for previous research [59]. For APT, the APT Integrity Scale has been developed for this study. For ST, the Treatment Integrity Scale will be used, based on previous research [11]. To assess competence, a scale for SPSP will be used that was developed for previous research [59] on the basis of instruments used in other psychodynamic trials [13, 75]. For ST, the Schema Therapy Competence Scale (STCS-I-1, [Young JE, Fosse G: Schema therapist competency scale (STCS-I-1), Unpublished document available on https://www.isstonline.com]) will be used and for APT, competence will be assessed by the ATOS therapist [31].

#### Treatment retention

In case of dropout of treatment, the therapist completes a questionnaire that assesses the reasons for dropout. The research assistant invites the patient for an exit interview and motivates the patient for completing additional assessments that were scheduled at the beginning of treatment. Patients are considered early completers if both patient and therapist consider the treatment as being successful. Another possibility is a ‘push out’ of the treatment program for various reasons, e.g. if commitment problems persists, treatment has negative effects or if the safety of therapists is endangered in other ways.

### Participant timeline {13}

#### Sample size {14}^3^

First, the required sample size was calculated to compare the three types of active treatments. Three pairwise comparisons will be conducted between the active treatment arms (ST vs. APT, ST vs. SPSP; APT vs. SPSP). We want to detect a medium effect size (*f*=0.11) in a pairwise comparison of pre-post change between two active treatment arms, equivalent to *f*(*v*)=.2460 and Cohen’s *d*=.492, with Bonferroni-corrected *p* = 0.0167, a power of 0.80 and within-person correlation coefficient=0.60, for which 88 patients are needed per arm. Because three types of therapies will be compared, 3 × 88 = 264 participants are needed to perform three pairwise comparisons. We chose a medium effect size (f=0.11^4^) since this would also be a clinically meaningful difference, as opposed to a smaller effect size.

The correlation of 0.60 is an educated guess of the correlation between repeated assessments based on the previous RCTs the authors conducted.

The final analysis will be conducted with mixed regression on time series: the change in slope over time between two treatments. Because power analysis for mixed regression are complex and based on complex assumptions about the covariance-structure, a simplified approach was chosen by conducting a power analysis on the pre-post change.

### Recruitment {15}

Patients are preselected for the NPI by the central admission department of Arkin Mental Health Care. This department performs a triage based on information of referrers and if necessary a telephonic screening, to select patients who need specialized care for personality pathology. One of the main inclusion criteria for referral to the NPI is the presence of at least one (or more) unsuccessful treatment(s) in specialized care in the past. Annually, the NPI receives over 2000 new applications Almost 50% of the patients is diagnosed with a Cluster-C PD or otherwise specified PD with predominantly Cluster-C traits. We expect around 600 patients to be eligible per year. Of these patients, 50% is expected to enrol in a second RCT (group psychotherapy for Cluster-C PD). Of the remaining 300 patients, we will need 25% of the eligible patients in order to end the trial on time. The inclusion period is estimated at 2.5 to 3 years.

**Fig. 1:**
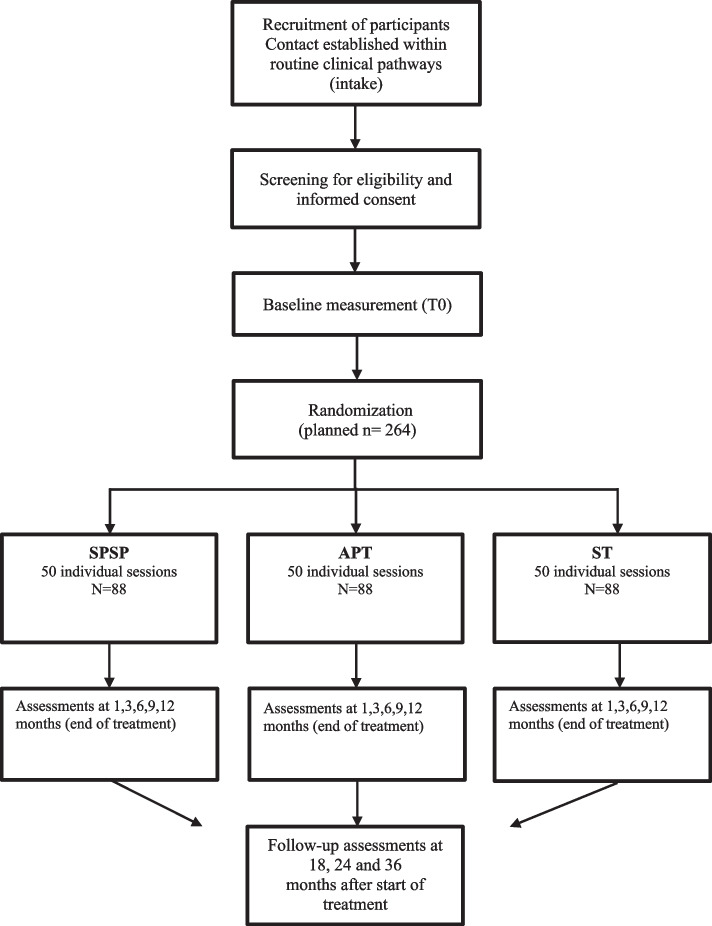
Flow chart of the study design

During intake, patients will be diagnosed using the SCID-5-P. The SCID-5-P will be assessed by the intaker. At the end of the intake procedure, the patients who are diagnosed with a Cluster-C PD (avoidant, dependent or compulsive) or another specified PD with predominantly Cluster-C traits, will be provided with both written and face-to-face information about FORCE by the intaker. If a patient is willing to participate, the therapy setting for individual or group therapy is decided after a protocolized shared decision making procedure. In this decision procedure, the first step is to check if group therapy is indicated. If so, the patient will be referred for the RCT on group therapy (G-FORCE, (Van den Heuvel, B, Dekker, JM, Daniëls, M, Van, LH, Peen, J, Bosmans, J, et al: G-FORCE: The effectiveness of group psychotherapy for Cluster-C personality disorders: a pragmatic RCT comparing two forms of schema group therapy and psychodynamic therapy, Manuscript submitted)). When a contra-indication for group is applicable, or patients prefer individual therapy, they are selected for I-FORCE. The intaker informs the research assistant who invites the patient for a screening procedure. The informed consent will be sent to the patient at least 1 week before the screening procedure, and if the patient agrees, they will be asked to sign the informed consent (see Fig. 1, the patients’ flow of I-FORCE).

### Who will take informed consent? {26a}

The first screening for eligibility is done by the intake clinician when the patient enrols at the NPI. After a positive screening, the research assistants will obtain informed consent from the potential trial participants.

### Additional consent provisions for collection and use of participant data and biological specimens {26b}

Not applicable. The informed consent covers all necessary information.

## Assignment of interventions: allocation

### Sequence generation {16a}

When patients are eligible for participation, the informed consent is signed and the baseline assessment is completed, they will be randomized to SPSP, APT or ST by the independent statistician, using computerized random assignment.^5^ Randomization of patients will be pre-stratified according to type of PDs (avoidant, obsessive, dependent, otherwise specified PD) and location (Amsterdam or Amersfoort). According to the treatment capacity, the randomization rate between SPSP, APT and ST can be adjusted if necessary.

### Concealment mechanism {16b}

Allocation concealment will be ensured, as randomization is computer-generated and will only be done after the patient has signed the informed consent.

### Implementation {16c}

Allocation sequence will be done by an independent statistician with an 1:1:1 allocation, using a computer script performing block randomization. Enrolment and assignment will be done by the research assistants.

## Assignment of interventions: blinding

### Who will be blinded {17a}

Blinding of therapists and participants is not possible due to the nature of the study. The collection of treatment session recordings will include contact with therapists. The baseline assessments will be done by a research assistant blind for treatment allocation. The other assessments will be performed by independent research assistants blind to treatment. Blinding is done using password authorization for randomization.

### Procedure for unblinding if needed {17b}

Participants and therapists are not blinded; therefore, a procedure for unblinding is not applicable.

## Data collection and management^6^

### Plans for assessment and collection of outcomes {18a}

Data will be derived from electronic patient records and collected with an electronic Case Report Form (eCRF) using ACCESS. Patients will use an online survey (NetQ) to answer questionnaires. Interviews will be coded and results will be stored in NetQ. Audiotapes will be stored in MSTeams in a separate folder protected with a password. All data acquired during the study will be anonymized and saved in a study folder on our protected research server. Only the study team has access to this specific study folder.

### Plans to promote participant retention and complete follow-up {18b}

The patients will receive extensive information about the study set-up and requirements during the recruitment. The importance of completion of the follow-up will be stressed. Patients are allowed to stop at any time during the study and are not obliged to give a reason to discontinue. Questionnaires are completed using an online survey, and therefore patients can do this at any convenient moment plus minus 2 weeks from the assessment time-points. All patients are reminded throughout the study to fill out the questionnaires during study visits. Throughout the follow-up period, the researchers will check responses and if necessary contact patients for completion of their follow-up. Participants will receive a gift voucher of 20 euros after completing the assessment at 24 months and another 20 euros after the final follow-up measurement has been completed.

### Data management {19}

We use Case Report Forms (eCRF) in an online manner (digital platform NetQ), which will allow standardized data capture, as well as facilitate typing, versioning or uploading of documents. In addition, each assessment will have a standardized operational procedure (SOP) to increase internal consistency. Such SOPs determine who may conduct the assessment (either online or face-to-face), evaluation steps and standardized communication with research participants.

### Confidentiality {27}

Research data will be stored using a study identification code for each participant. The key of these numbers will only be available to the principal investigator and the independent statistician of Arkin Mental Health Care and will be documented and safeguarded by the principal investigator according to research guidelines after completion of the study. No patient identification details will be reported in publications.

### Plans for collection, laboratory evaluation and storage of biological specimens for genetic or molecular analysis in this trial/future use {33}

Not applicable, no biological specimens will be collected or analysed in this research.

## Statistical methods^7^

### Statistical methods for primary and secondary outcomes {20a}

#### Primary study parameter(s)

##### Treatment response: ADP-IV

All analyses will be conducted according to the intention-to-treat principle. Primary and secondary outcomes are analysed with Linear Mixed Models, with random effect of site (if estimation allows). The primary outcome measure is the change in the total ADP-IV score using the dimensional scoring algorithms [86]. Change in the primary outcome measure and the relative effectiveness of the three treatments will be analysed using mixed regression so that all available data are used, and taking into account the levels of participant and time. The addition of random levels for therapist, to adjust for possible therapist effects, will be tested. The underlying distribution of the mixed regression model will be determined based on the distribution of residuals (e.g. normal, gamma, negative binomial). Per-protocol analyses will also be conducted to test for robustness and sensitivity.

##### Differential treatment response: four potential moderators

To gain more insight into differential treatment response, we will first examine which of the potential predictors, nominated in interviews by expert clinicians and based on the literature (see Table 3), actually predict (differential) treatment response. We will adopt the Personalized Advantage Index analysis developed by DeRubeis [27] that was previously used in the research group of the Principal Investigator [19, 20]. We will first build four univariate regression models for each predictor, with the predictor and separate variables for the interaction with the three interventions (moderators). We will then build a multivariable regression model with all four predictors and separate variables for the predictors and their interaction with the three interventions, to examine the relative contribution of each potential predictor or moderator. The outcome variable is the ADP-IV. In addition, we plan to also do a data-driven approach, in which we use machine learning techniques to select the moderators that go into the Personalized Advantage Index algorithm.

##### Mechanisms of change

It is hypothesized that the interventions exert a positive effect on the ADP-IV through their impact on the treatment-model-specific (i.e. insight ratings in SPSP, change in affect /defences/self-esteem ratings in APT and schema mode ratings in ST) and non-specific or common (i.e. alliance) mechanisms of change. To identify non-specific and specific mechanisms of change and the strength of the factors involved, both multilevel mediation models and structural equation models will be used. These analyses are based on the Latent Change Score models for mediation used in the research group of the Principal Investigator [19, 20].

#### Secondary study parameter(s)

##### (Differential) treatment response: secondary study parameters

We will examine change in the secondary outcome measures in the three treatments using mixed regression analysis. The secondary outcome measures include diagnostic status, reliable change and recovery, personality functioning, general functioning, general psychopathology, quality of life and happiness. As the PD severity indices AVPDSI, DPDSI and OCPDS vary per primary PD, they will be first standardized before they are analysed in the complete sample. This analysis will be controlled for primary PS, to account for possible differences in sensitivity to change. The underlying distribution of the mixed regression model will be determined based on the variable type (scale, nominal) and the distribution of residuals (e.g. normal, gamma, negative binomial). In addition, preliminary analyses will test differential treatment retention using survival analysis and mixed logistic regression. Medication confounds will be examined.

##### Cost-effectiveness

The cost-effectiveness analysis (CEA) will be conducted from a societal perspective. Within the CEA, the difference in societal costs (measured by the TiC-P at baseline and after 6, 12, 18, 24 and 36 months) generated by patients in the three treatments will be related to the difference in clinical effects (measured with the ADP-IV and quality-adjusted life-years based on the EQ-5D-5L) over the course of 36 months. Missing cost and effect data will be imputed using multiple imputation. Mixed model regression analyses will be used to estimate cost and effect differences between groups. Bootstrapping with 5000 replications will be used to estimate 95% confidence intervals around cost differences and the uncertainty surrounding the incremental cost-effectiveness ratios (ICERs). Uncertainty surrounding the ICERs will be graphically presented on cost-effectiveness planes. Cost-effectiveness acceptability curves [36] will also be estimated. Adjustment for confounders and effect modifiers will be done if necessary.

The statistical analyses described above are still under development and examples of possible methods. At the time of the analyses, we will use the optimal method for analysing the data.

### Interim analyses {21b}

There are no interim analyses planned.

### Methods for additional analyses (e.g. subgroup analyses) {20b}

For the three primary cluster-C PDs (avoidant, dependent, obsessive-compulsive) and the otherwise specified PD with predominantly cluster-C traits, subgroup analyses are planned.

### Methods in analysis to handle protocol non-adherence and any statistical methods to handle missing data {20c}

The primary outcome will be assessed using an analysis. Missing data will be reduced to a minimum by using the appropriate measures described above.

### Plans to give access to the full protocol, participant-level data and statistical code {31c}

The full protocol can be made available by the corresponding author upon reasonable request and in agreement with the research collaboration.

Because of the sensitivity of the data and the strict policies for data security, we will not share our participant-level data with external parties or in external repositories.

## Oversight and monitoring

### Composition of the coordinating Centre and trial steering committee {5d}

This is a mono-centre study designed, performed and coordinated in Arkin/NPI. Day to day support for the trial is provided by:

- Principal investigator: takes supervision of the trial and medical responsibility of the patients.
- Data manager: organizes data capture, safeguards quality and data.
- Study coordinator: trial registration, coordinates the study, annual safety reports.
- Research assistants: identify potential recruits, take informed consent, conduct interviews and ensure follow-up according to protocol.
- The research committee meets biweekly. There is no trial steering committee or stakeholder and public involvement group.

### Composition of the data monitoring committee, its role and reporting structure {21a}

In agreement with the ethical committee of the VU, a DSMB has not been appointed for this study.

### Adverse event reporting and harms {22}

There are no direct risks involved for patients enrolled in this study. All patients will receive an evidence-based treatment (i.e. SPSP, APT or ST). Adverse events are defined as any undesirable experience occurring to a subject during the study, whether or not considered related to the experimental intervention. All adverse events reported spontaneously by the subject or observed by the investigator or his staff will be recorded. Serious adverse events will be reported within 24 h to the principal investigator.

### Frequency and plans for auditing trial conduct {23}

No auditing of trial conduct is planned.

### Plans for communicating important protocol amendments to relevant parties (e.g. trial participants, ethical committees) {25}

Important protocol modifications will be communicated with the ethical committee of the VU Amsterdam and if necessary with the therapists and the research participants.

### Dissemination plans {31a}

The trial results will be offered for publication in a peer-reviewed journal. The article will be shared with the therapists and participants of the trial.

Because of the use of privacy sensitive information, the database will not be shared in a public repository.

## Discussion

This article presents the design of I-FORCE, a pragmatic clinical trial comparing the effectiveness of three individual psychotherapies for Cluster-C PDs, short-term psychodynamic supportive psychotherapy (SPSP), affect phobia therapy (APT) and schema therapy (ST). I-FORCE responds to the need to broaden the evidence for the treatment of Cluster-C PDs, a group of patients with high prevalence rates in the general and clinical population.

In the current literature, the number and quality of the conducted studies on Cluster-C PD is fairly low, with small sample sizes [56]. This relatively large RCT will extend the evidence considerably. The broad range of outcome variables will give insight in effectiveness on symptomatic, descriptive and of underlying characteristics of personality pathology. By investigating predictors and moderators, this study moves from general effectiveness to a more tailored insight into differential effects of the treatments and interventions. Repeated assessments of mediators during treatment offer us the option to study the temporal relationships between potential mediators and outcome. With the 2-year follow-up period of this study, we can provide insight in the sustainability of gains of treatment, both at a level of symptoms and personality.

In addition, this study will provide insight into the effectiveness and feasibility of Cluster-C tailored protocols. For the three treatments of this study, well-defined protocols are implemented, addressing the phases of treatment, interventions per phase, number of sessions, intensity of treatment and directions for the therapeutic stance. All protocols have a personalized character, offering a balance between directiveness of the interventions on the one hand and the option for adjustment to the personal needs of the patient on the other hand.

### Strengths

To the best of our knowledge, this is the first study comparing psychodynamic treatments for the Cluster-C population head-to-head to ST, a treatment that has proven its efficacy for this population within one earlier RCT [11]. This RCT will contribute to the body of evidence of the effectiveness of time-limited individual PD treatments.

Another strength is the pragmatic nature of this study. By staying close to the clinical reality and daily practice (e.g. allowing add-on interventions, medication use, no preselection of therapists, no intensive central supervision), we can include a wide range of Cluster-C patients and study the effectiveness in an ecologically valid environment. This implies that the sample is aimed to be representative of the clinical field and results could be generalized to daily mental health practice.

A final strength is that the pragmatic nature of this study is balanced by the deliberate attention for the quality and adherence of the delivered psychotherapies. This is ensured by deployment of qualified therapists, who have attended treatment-specific training and attend peer supervisions frequently throughout the treatment. Adherence and performance will be checked by well-defined treatment adherence checks.

### Limitations

There are several limitations to consider. First, in the design of the study, no inactive control group was added. This means that the influence of the natural course of variation over time cannot be ruled out. We did not include a no-treatment comparison group due to ethical issues involved in withholding treatment for this population seeking specialized PD care.

Second, by including a wide range of patients, Cluster-C PDs and the group of patients with an otherwise specified PD diagnosis, the sample might be more heterogeneous than in studies only including full Cluster-C PDs. However, the inclusion criterion of a predominance and a minimum of five Cluster-C traits and the exclusion of (subthreshold) cluster-A and B PD, ensures that patients with predominantly Cluster-C pathology are included. By adding the otherwise specified PD patients, we expect to better adapt to the clinical practice, where the prevalence of full Cluster-C diagnosis is less common than mixed Cluster-C diagnosis.

Third, we did not adjust the sample size for dropout, which might have a negative impact on the power. However, we expect mixed regression with multiple measurements between pre- and posttreatment will compensate for the potential loss of power due to dropout.

Lastly, there might be some overlap between the delivered psychotherapies. APT and ST both are integrative therapies, using elements of psychodynamic, experiential, cognitive and behavioural therapy. Also, APT and SPSP overlap in some of their interventions and in their focus on developmental needs. It has been suggested that general common factors, and in particular the therapeutic relationship, are the most relevant working mechanisms for psychotherapy. Nevertheless, in practice, we think the approaches are perceptibly different at the level of applied concepts and techniques. In ST, this difference is seen in the focus on the mode model and experiential techniques (e.g. imagery rescripting and chair techniques), in APT in the attention for anxiety regulation, perception of the self and activation of affect. SPSP on the other hand is a more exploratory, supportive treatment, focussing on the levels of discourse used to gain insight. We therefore expect a difference in approach to be perceptible for both therapists and patients. This is checked by the treatment intervention checklist after every session and in the adherence part of the study. In addition, this study investigates if improvement on the therapy-specific working mechanisms differs between the three therapies or if they improve equally over all treatments.

In sum, this study aims to enlarge the body of evidence for the (cost)-effectiveness of psychotherapeutic treatment for Cluster-C PDs. Also, we will get a more in-depth insight into the differential effects of the various ingredients of the Cluster-C treatments. We hope to enhance optimization of the effectiveness of the therapies and enable more personalized treatment decisions for this neglected group of patients.

## Trial status

Recruitment started October 1st 2020. Recruitment is planned to be completed in October 2023.

## Data Availability

According to the Dutch General Data Protection Regulation, the data, datasets and results cannot be provided to external parties and will not be published on a public repository.

## Abbreviations

ADP: Assessment of DSM-IV Personality Disorders questionnaire
APT: Affect Phobia Therapy
AQ: Autism Questionnaire
AVPDSI: Avoidant Personality Disorder Severity Index
BEAQ: Brief Experiential Avoidance Questionnaire
BPDSI: Borderline Personality Disorder Severity Index
BSI: Brief Symptom Inventory
CCMO: Central Committee on Research Involving Human Subjects
in Dutch: Centrale Commissie Mensgebonden Onderzoek
DDPRQ: Difficult Doctor-Patient Relationship Questionnaire
DEPDSI: Dependent Personality Disorder Severity Index
DSMB: Data Safety Monitoring Board
CTSC-R: Client Task-Specific Change Measure-Revised
CTQ-SF: Childhood Trauma Questionnaire-Short Form
DPI-SF: Developmental Profile Inventory-Short Form
ICER: Incremental cost-effectiveness ratio
I-FORCE: Individual psychotherapy FOR Cluster-C Effect study
I-FORCE-TIL: I-FORCE-Treatment Intervention List
IPO: The Inventory of Personality Organization
MEAQ: Multidimensional Experiential Avoidance Questionnaire
NNS: Nederlandse Narcisme Schaal
OCPDSI: Compulsive personality disorder severity index
PD: Personality disorder
RCT: Randomized clinical trial
RSES: Rosenberg Self-Esteem Scale
SCID-II: Structured Clinical Interview for DSM-IV Axis II Disorders
SCID-5-P: Structured Clinical Interview for DSM-5 Personality Disorders
SMI: Schema Mode Inventory
SPSP: Short-term Psychodynamic Supportive Psychotherapy
ST: Schema therapy
TAU: Treatment-as-usual
TiC-P: Treatment Inventory Cost in Psychiatric Patients
STCS: Schema Therapy Competence Scale
WAI: Working Alliance Inventory
WHODAS: World Health Organization Disability Assessment Schedule

## Footnotes

1 The in- and exclusion criteria are similar to the criteria described in the protocol article of the G-FORCE study (Van den Heuvel, B, Dekker, JM, Daniëls, M, Van, LH, Peen, J, Bosmans, J, et al: G-FORCE: The effectiveness of group psychotherapy for Cluster-C personality disorders: a pragmatic RCT comparing two forms of schema group therapy and psychodynamic therapy, Manuscript submitted).

2 Due to equal measurements in I-FORCE and G-FORCE, the description of the measurements is similar in both manuscripts (Van den Heuvel, B, Dekker, JM, Daniëls, M, Van, LH, Peen, J, Bosmans, J, et al: G-FORCE: The effectiveness of group psychotherapy for Cluster-C personality disorders: a pragmatic RCT comparing two forms of schema group therapy and psychodynamic therapy, Manuscript submitted).

3 The description of the calculation of the sample size is identical to the description in the G-FORCE protocol paper (see Van den Heuvel, B, Dekker, JM, Daniëls, M, Van, LH, Peen, J, Bosmans, J, et al: G-FORCE: The effectiveness of group psychotherapy for Cluster-C personality disorders: a pragmatic RCT comparing two forms of schema group therapy and psychodynamic therapy, Manuscript submitted).

4 This effect size *f* is used in GPower for the within-between interaction and is not the equivalent of the between effect size *f*. We therefore reported the equivalent effect sizes according to Cohen (i.e. *f*(*v*)=.2460), and Cohen’s *d*=.492—which are approximately medium.

5 The procedure of randomization has been changed during the process of inclusion. Previously, randomization was performed before the baseline assessment. Any possible effect of this change in procedure will be researched.

6 The data collection and management for I-FORCE uses the same procedures as the G-FORCE study. Therefore, the description in this article is similar to the G-FORCE protocol article (see Van den Heuvel, B, Dekker, JM, Daniëls, M, Van, LH, Peen, J, Bosmans, J, et al: G-FORCE: The effectiveness of group psychotherapy for Cluster-C personality disorders: a pragmatic RCT comparing two forms of schema group therapy and psychodynamic therapy, Manuscript submitted).

7 The statistical methods of I-FORCE are similar in design and procedure to G-FORCE. Therefore, the description of the statistical methods is similar to the description in the I-FORCE protocol paper (see Van den Heuvel, B, Dekker, JM, Daniëls, M, Van, LH, Peen, J, Bosmans, J, et al: G-FORCE: The effectiveness of group psychotherapy for Cluster-C personality disorders: a pragmatic RCT comparing two forms of schema group therapy and psychodynamic therapy, Manuscript submitted).
